# Tissue Engineering Considerations in Dental Pulp Regeneration 

**Published:** 2013-12-24

**Authors:** Ali Nosrat, Jong Ryul Kim, Prashant Verma, Priya S. Chand

**Affiliations:** a * Department of Endodontics, Prosthodontics and Operative Dentistry, School of Dentistry, University of Maryland, Baltimore, MD, USA; *; b *Iranian Center for Endodontic Research, Shahid Beheshti University of Medical Sciences, Tehran, Iran*

**Keywords:** Growth Factor, Progenitor Cells, Pulp Regeneration, Scaffold, Stem Cell, Tissue Engineering, Transplantation

## Abstract

Regenerative endodontic procedure is introduced as a biologically based treatment for immature teeth with pulp necrosis. Successful clinical and radiographic outcomes following regenerative procedures have been reported in landmark case reports. Retrospective studies have shown that this conservative treatment allows for continued root development and increases success and survival rate of the treated teeth compared to other treatment options. Although the goal of treatment is regeneration of a functional pulp tissue, histological analyses show a different outcome. Developing predictable protocols would require the use of key elements for tissue engineering: stem cells, bioactive scaffolds, and growth factors. In this study we will review the evidence based steps and outcomes of regenerative endodontics.

## Introduction

Treatment of immature necrotic teeth is a challenge in endodontics. Arrested root development following pulp necrosis can lead to weak root structure with thin dentinal walls, which makes the tooth susceptible to fracture and reduces its survival rate [1]. Historically, multiple visit apexification with calcium hydroxide was the treatment of choice in necrotic immature teeth to induce formation of an apical hard tissue barrier [2]. While this approach was predictable and successful , long term use of calcium hydroxide has several disadvantages that includes multiple treatment appointments, probable recontamination of the root canal system, and increased brittleness of root dentin which might increase the risk of cervical root fractures [3, 4]. Apical barrier technique or root canal obturation using bioactive cements was introduced as an alternative technique [5-7]. Although these techniques have reasonable success rates in terms of periapical disease healing [8-10], none of the aforementioned treatment approaches result in continued root development and strengthening of root structure, and therefore, the long-term structural integrity of the teeth may be compromised.

Recently, a new approach has been introduced which is aimed at regeneration of the pulp-dentin complex and inducing root development in immature teeth with necrotic pulps [11, 12]. The treatment is based on the presence of viable *Stem Cells from Apical Papilla* (SCAP) which are the source of primary odontoblasts in normal root development process [13]. Under ideal circumstances such as absence of bacteria and necrotic tissues, presence of an appropriate scaffold, and a tight coronal seal, these cells might be able to repopulate the root canal space and reestablish the pulp-dentin complex.

In a recent protocol published by American Association of Endodontists (AAE), the procedure for regenerative endodontic treatments in immature teeth with necrotic pulps, has three separate steps during at least 2 appointments [14]. The *first* *step* is root canal disinfection with sodium hypochlorite. Although the AAE’s recommended concentration for NaOCl is 1.5%, there are several successful clinical reports of using higher concentrations of NaOCl in regenerative endodontic treatments including 6% [15, 16], 5.25% [17-19] and 2.5% [20-22]. The next level of the disinfection consists of root canal dressing with an antibiotic [17, 23] or calcium hydroxide paste [20, 24]. The goals for this step are to control the patient’s acute symptoms (if present) and to reach the highest possible level of disinfection to make the root canal space an appropriate environment for regenerating the pulp-dentin complex. The most prevalent antibiotic used for this purpose is an equal mixture of metronidazole, minocycline, and ciprofloxacin, called the Triple Antibiotic Paste (TAP). Studies on TAP have shown its efficacy in disinfection of the root canal space [25] and deep layers of dentin [26]. Although the most efficient antibiotic against endodontic bacteria in this mixture is minocycline [27], there are clinical reports of successful disinfection of the root canals by using double antibiotic paste (metronidazole and ciprofloxacin) [12, 28] and modified TAP (metronidazole, ciprofloxacin, and cefaclor) [29]. A recent review on the cases treated from 2004 to 2012 showed that the aforementioned disinfection protocol is a successful strategy which has been documented with radiographic healing of the periapical disease in all reported cases [30]. The *second step *is preformed during the second appointment which involves removing the canal dressing, a final flush with 17% ethylenediaminetetraacetic acid (EDTA) and inducing a blood clot inside the root canal space by irritating the periapical tissues by means of a sterile hand file. Use of EDTA at this step causes release of sequestered growth factors form dentinal walls which promotes the proliferation and differentiation of SCAP [31]. Another potential source of growth factors in this treatment protocol is the release of growth factors following disintegration of platelets in blood clot [11]. The blood clot also acts as a protein-rich scaffold and also delivers SCAP into the root canal space. One study showed that the level of markers for human stem cells in the blood clot formed inside the root canal space is up to 600 times higher than the same markers in peripheral blood [32]. Recent reports have shown that platelet-rich plasma (PRP) can also be used as a scaffold at this step [33, 34]. As the *third step*, the blood clot has to be sealed by a biocompatible material followed by restoration of the access cavity. The most recommended biomaterial for sealing the pulp space is mineral trioxide aggregate (MTA) [35]. One study documented successful use of calcium enriched mixture (CEM) cement as a sealing material on blood clot [23].

Retrospective outcome studies show that this new treatment protocol for immature teeth with pulp necrosis causes increase in root length and root wall thickness [36, 37]. Use of TAP as an intra-canal medicament is associated with significantly greater increase in root wall thickness compared to calcium hydroxide [36]. A recent study by Jeeruphan *et al.* [37] showed that teeth treated by regenerative endodontic treatment have significantly higher survival rates and radiographic healing rates (of periapical lesions), compared to teeth treated by apexification with calcium hydroxide. Although the success and survival rate of cases treated with regenerative endodontic procedures were higher than cases treated with MTA apical plug, the differences were not significant [37]. Based on these outcome studies, this new protocol might be a better option for treatment of immature teeth with necrotic pulp regardless of the type of tissues formed inside the canal after treatment. However, randomized clinical trials, which offer the highest level of evidence, are still awaited.

Although there are several reports of favorable outcomes of this treatment in the literature, this new approach can potentially cause unfavorable outcomes, which need to be addressed:

***a) Discoloration***: Tooth discoloration following regenerative endodontic treatment is a serious problem. As showed by Kim *et al.* the main cause of tooth discoloration is minocycline in TAP [38]. Other studies have shown that TAP has the highest discoloration potential among any other endodontic material [39]. Using double antibiotic paste [12] or modified TAP [29] might be the solution to prevention of discoloration caused by minocycline. In addition, presence of MTA might be another source for discoloration [18, 30] which can be prevented by using alternative tooth-colored bioactive materials like CEM cement over the blood clot [23, 40] which has been shown to be biocompatible with acceptable sealing ability [41-43].

***b) Unfavorable or no root development***: Root development in immature teeth includes increase in root length, increase in wall thickness, and closure of the apex (root maturation). Lack of the aforementioned criteria in root development has been reported previously which can be considered as a potential unfavorable outcome [18, 30, 44]. A review on dental history of cases treated with regenerative endodontic treatments revealed a relation between long-term pulp necrosis before treatment (>6 months) and poor outcomes in terms of root development [30]. This might be due to the presence of well-established bacterial biofilms on the dentinal walls which would be hard to remove with minimal or no instrumentation as recommended in the AAE’s protocol. Also, damage to the SCAP or Hertwig’s epithelial root sheath due to long-term periapical infection or dental trauma have been suggested as other reasons [12, 30]. Interestingly, all of the reported cases with unfavorable or no root development have shown complete radiographic healing of the periapical lesions [30]. For traditional endodontic treatment, lowering the bacterial load and preventing bacterial access to periapical tissues might be conductive to healing. However, a higher level of disinfection is required for pulp regeneration [45]. Further, it is worth mentioning that in younger teeth, bacteria can penetrate into more dentinal tubules and advance deeper compared to older teeth [46]. Disinfection of the root canal spaces of immature teeth is therefore quite challenging, and there is a need to develop more effective antimicrobial regimens to create an environment conducive to predictable regeneration of the pulp-dentin complex and root development. 

***c) Empty root canal spaces (no tissue regeneration):*** The probability of empty root canal space after regenerative endodontic treatment in cases with poor or no root development was first discovered by Lenzi and Trope [47]. They treated two immature maxillary central incisors with pulp necrosis and periapical lesions. Teeth were in different stages of development. The one with shorter root and wider apex had larger periapical lesion and showed no root development 21 months after treatment. They hypothesized that the physically weak blood clot might have disintegrated after treatment, leaving the root canal space empty. However, they documented complete healing of the periapical lesion on periapical radiographs and 3D images. Nosrat *et al.* documented empty root canal spaces clinically in two maxillary central incisors 6 years after regenerative treatment [30]. Both teeth showed complete healing of the periapical lesions and radiographic signs of apical closure (root maturation) without increase in root length or wall thickness. Since the exact criteria for success of the treatment has not been determined yet, these findings might not be considered as “clinical failures” but show that the outcome of the current protocol for pulp regeneration might be unpredictable. 

## Histological outcomes of animal studies

Animal studies on the protocol for pulp regeneration have documented the formation of new hard tissues on root dentinal walls and apical closure in radiography and histology. They also showed new tissues growing into the root canal space [48-50]. All of these studies have been conducted on dog models. Thibodeau *et al.* evaluated the effect of blood clot and a soluble collagen scaffold on the outcome of the treatment [48]. They showed that the amount of vital tissues inside the root canals and the amount of hard tissue deposited on the dentinal walls were not different in the presence or absence of blood clot inside the root canal space. In addition, presence of a soluble collagen scaffold did not improve the results. Detailed histological evaluation of the same specimens by Wang *et al.* revealed that there were three types of tissues formed inside the root canals following the treatment: *cementum-like tissue* which was responsible for increase in root length and thickness, *bone-like tissue*, and *periodontal ligament (PDL) like tissue* inside the canal space [49]. da Silva *et al.* showed that the vital tissue formed inside the root canal space after treatment is generally an ingrowth of periodontal connective tissue to the root canal space [50]. An interesting finding in this study was presence of inflammation in almost all of the specimens in experimental groups. They also showed that using EndoVac with NaOCl for root canal irrigation would be a better option than irrigation with NaOCl plus TAP dressing. Yamauchi *et al.* attempted to perform pulp regeneration in a dog model by adding an insoluble collagen scaffold to the blood clot and using 17% EDTA [51]. EDTA was used as a demineralizing agent to release growth factors embedded in dentinal walls and expose dentine matrix and therefore, promote differentiation of mesenchymal cells. Immuno-histochemical analysis of the tissues formed after the treatment showed that the hard tissue formed on the dentinal walls was a cementum-like tissue with different organization and maturation of collagen fibers. 

All of these animal studies showed that the recent protocol of pulp regeneration is not predictable in terms of type of tissues formed inside the root canal and on the dentinal walls. However, none of these animal studies investigated the presence of stem cells in the apical papilla after inducing the periapical infection, or in the blood clot.

## Histological reports of human teeth

The first histological report of outcome of pulp regeneration in human teeth was done by Torabinejad and Faras [52]. In this study root canal treatment was performed in a maxillary premolar which had received regenerative endodontic treatment by using PRP as scaffold 14 months before. Because of the method of tissue sampling they were not able to provide information about pulp-dentin complex and the hard tissue deposited on the root canal walls. They showed presence of a pulp-like connective tissue inside the canal. Shimizu *et al.* performed histological evaluations on a maxillary central incisor three and a half weeks after treatment [53]. The tooth was extracted because of an oblique enamel-dentin fracture due to a traumatic impact. They showed that the tissue inside the root canal was a loose connective tissue similar to an immature pulp containing spindle-shaped fibroblasts and mesenchymal cells. Another report on the histological outcome of the treatment in human teeth was done by Martin *et al.* [54]. They performed regenerative endodontic treatment in a mandibular molar by using PRP in distal canal and blood clot in mesial canals. The tooth was extracted 25 months after the treatment because of an oblique fracture of the lingual cusps. The mineralized tissue formed on the root canal walls was cementoid/osteoid tissue and the root canal spaces were filled with vital connective tissue and blood vessels. Regardless of the scaffold type, they showed that there was no pulp tissue inside the roots.

All in all, the aforementioned data shows that although the recent protocol for pulp regeneration has higher success rate and survival rate compared to other methods of treatment for immature teeth with pulp necrosis, the biological outcome of the treatment is still not predictable and it may not be true pulp regeneration. Part of the problem might be related to the disinfection strategies. A study by Trevino *et al.* showed that full strength NaOCl is toxic to SCAP and prevents repopulation of SCAP on dentinal walls [55]. Investigation of the ability of dental pulp stem cells (DPSCs) to adhere to dentin after being exposed to different irrigants revealed that irrigation with NaOCl was related to a lower degree of cell adherence [56]. In addition, denaturation of dentinal proteins by NaOCl interferes with differentiation of stem cells in contact with dentin [57]. Routine antibiotic dressings with creamy consistency might be another threat to pulp regeneration. A recent study by Ruparel *et al.* showed that TAP with creamy consistency (1000 mg/mL) would be extremely toxic to SCAP [58]. The study revealed that a concentration of 0.01 to 0.1 mg/mL would be conducive to SCAP survival. A low concentration of antibiotic is a watery solution which would not be stable inside the root canal space and therefore, a biocompatible carrier has to be used to hold the dressing for a few weeks. Besides, the antibacterial efficacy of antibiotics in this low concentration is another concern which deserves further studies.

It has been proposed that the way forward is to develop therapies based on sound tissue engineering principles using a triad of stem cells, growth factors and bioactive scaffolds [11]. There are advantages in such attempts to direct or orchestrate the regenerative process including futuristic possibilities such as being able to custom design a pulp with appropriate immune response, vitality and sensibility. Controlled regeneration would also avoid unfavorable outcomes, and applications for treating mature teeth could be developed. The regenerated pulp tissue could potentially be directed to produce a dentin overlay precisely where needed to restore damaged tooth structure, or to increase root length and root thickness.

## Potential role of stem cells

During wound healing after pulp exposure, the undifferentiated mesenchymal cells migrate to the site of exposure from the deeper regions of the pulp, and replace degenerated odontoblasts [59]. An extension of this concept is that in regenerative endodontic treatment of cases with pulp necrosis, stem cells are required to achieve the goal of replacing the diseased pulp tissue with a regenerated healthy pulp that would continue normal dentinogenesis. The ideal healthy pulp would be capable of a robust immune response, and exhibit vitality through blood supply and sensibility through nerve supply. Vascular and neural regeneration require stem cells to differentiate into respective progenitor cells.

Stem cells have several unique characteristics. They exist as undifferentiated cells and maintain this phenotype until they are exposed to the appropriate signals. They have extensive self-replication capacity, and can maintain themselves throughout the life of an organism. Stem cell populations are found in most adult tissues. Adult stem cells have more restricted differentiation potential than embryonic stem cells, but they avoid the ethical constraints of the latter and are thus well suited for research and clinical therapies [60]. 

Current clinical protocols in regenerative endodontics are probably utilizing indigent stem cells to form progenitor cells, but they make no attempt to orchestrate the regenerative process, and the histologic character of the tissues formed is quite variable. These indigent stem cells come from the following sources:

***a) Stem cells of the apical papilla (SCAP)***

These are a population of mesenchymal stem cells (MSCs) residing in the apical papilla of incompletely developed teeth. SCAP appear to be the source of primary odontoblasts that are responsible for the formation of root dentin, whereas *dental pulp stem cells* (DPSCs) are likely the source of replacement odontoblasts [13]. When transplanted subcutaneously into immuno-compromised mice, they have been shown to differentiate into odontoblasts and form dentin [61]. Because of its apical location, the apical papilla has collateral circulation and this enables it to survive during the process of pulp necrosis. The evoked bleeding step in current clinical protocols for pulp regeneration triggers the significant accumulation of stem cells into the canal space [32]. These cells are likely to be SCAP cells and might contribute to the regeneration of pulpal tissues.

***b) Dental pulp stem cells (DPSCs)***

Human DPSCs have self-renewal capability and multi-lineage differentiation potential *in vitro* [62, 63], including dentinogenic potential [64, 65]. Many cases being treated by regenerative endodontics have residual inflamed pulp tissue, and it has been shown that this tissue contains DPSCs, which retain tissue regeneration potential [66].

***c) Periodontal ligament stem cells (PDLSCs)***

PDL contains stem cell populations that can differentiate into cementoblasts or osteoblasts [67, 68]. PDLSCs exhibit osteogenic, adipogenic and chondrogenic capacity under defined culture conditions, but they don’t seem to be dentinogenic [69-71]. The evoked bleeding step in current clinical protocols may also lead to PDLSCs accumulation in the root canal, and they may be responsible for the bone-like tissues formed in the canal in some animal studies [72].

Several case reports have shown successful outcomes with regenerative endodontic treatment, in terms of lesion resolution and increase in root length and/or thickness [18, 23, 33]. In such cases when complete success is not achieved, conventional non-surgical root canal treatment or MTA apexification is still feasible and often made technically easier due to some degree of apical closure from the regenerative treatment. It has been shown that the public health impact of regenerative endodontics is limited unless its applications are expanded to treat teeth in adult patients with mature apices [73]. These patients have no SCAP cells, and the supply of indigent stem cells in the periapical areas may vary with the age of the patient and size of the periapical lesion. 

There are two major approaches to deliver stem cells into the root canal for pulp regeneration:

***i. Cell transplantation***

This involves direct delivery of autologous or allogeneic stem cells into the root canals. Studies in animals have shown promising results with this approach for regenerative endodontics [74-76]. There is still a need for good *in situ* models for human regenerative procedures, as they would be used on patients. The major roadblocks for clinical translation are immune rejection potential (for allogeneic cells), regulatory approval for using in patients, and high cost. Mesenchymal stem cells have reduced immunogenicity because they are able to avoid the immune response and they also make factors that suppress the immune response [77]. These stem cells home to injured tissues and modulate the inflammatory response through synergistic down-regulation of pro-inflammatory cytokines and up-regulation of pro-survival and anti-inflammatory factors [78]. Immunocompatible off-the-shelf allogeneic stem cells could potentially be used to formulate a viable commercial product to use for total dentin-pulp regeneration in endodontic practice. 

***ii. Cell homing***

This involves the use of chemotactic factors like Stromal Cell Derived Factor (SDF)-1 that can induce migration of stem cells from the periapical area into the root canal. Immune rejection is therefore not an issue. This has been investigated in animal models as an adjunct to stem cell transplantation, but not as a stand-alone technique [74, 79]. The regulatory approval process to use this technique in human patients would be much easier, and the cost much lower. However, the results may depend on the distance that cells need to migrate, so the longer the root length, the less favorable the prognosis would be. Also adult patients that have fewer or no indigent stem cells may not be candidates for such treatment, and it has been shown that these patients comprise the majority of the patient population in endodontic practices [73]. Large periapical lesions may require very long treatment times, because a high level of disinfection is a prerequisite for successful regeneration [45], and a long time would be needed to wait for lesion resolution so that a source of stem cells to become available for homing into the root canal space.

The first manufactured medicament based on allogeneic stem cell transplantation was recently granted regulatory approval to treat children suffering from graft-versus-host disease (GVHD). Stem cell banking of *Stem Cells from Exfoliated Deciduous teeth* (SHED) and DPSCs from extracted third molars has already been commercialized in anticipation of clinical therapeutic applications. Inflamed pulp tissues are currently discarded as medical waste, but they have been shown to contain stem cells with regenerative potential and may be considered as an alternative and more attainable source of DPSCs [66]. The timeline for translation of tissue engineering into clinical endodontic practice remains to be seen, but there already appears to be an explosion of basic science research in this field.

## Potential role of bioactive scaffolds

An ideal scaffold should have the following characteristics for successful tissue regeneration: capability for seeding of stem/progenitor cells or supplementation with growth factors, controlled biodegradability to be eventually replaced with natural tissue, adequate biomechanical properties, bioactivity to facilitate attachment to soft and hard tissues, biocompatibility with the host tissue, osteoinductivity and angiogenic potential.

The most frequently used scaffold in regenerative endodontics is a blood clot [35]. The fate of blood clot in apical area of root canal after conventional root canal treatment was explored by Nygaard Østby in the early 1960s [80]. The blood clot produced by platelet aggregation was gradually transformed into fibrous connective tissue over time even in cases where the initial diagnosis had been pulp necrosis with apical periodontitis. In the current AAE protocol for regenerative endodontics [14], blood clot induced by lacerating periapical tissue with overextended file is suggested as a scaffold and also as the way to bring SCAP into the root canal [81]. Following the seminal case report by Banchs and Trope, blood clot has been used in revascularization of immature permanent teeth [17], regardless of the differences in disinfection protocol such as concentration of sodium hypochlorite and type of antibiotics [17, 23, 29]. The evoked bleeding from apical papilla into root canal showed significant accumulation of transcripts for the stem cell markers CD73 and CD105, compared with levels found in the systemic blood, which suggests that these cells will contribute to the regeneration of pulp tissue after disinfection of the root canal space [32]. It is recommended to use local anesthetics without vasoconstrictors in order to induce sufficient amount of bleeding into the root canal [18]. However, lack of bleeding or insufficient bleeding even after using plain local anesthetics has been reported [23, 24] and was associated with poor root development in some cases [23]. The absence of a blood clot has also been implicated in unsuccessful outcomes of regenerative endodontics in an animal study [48]. The use of PRP was suggested when it is difficult to produce periapical bleeding by overextending a file [33]. In an animal study comparing blood clot and PRP for pulp regeneration, there was no difference between the groups with respect to the presence of vital tissue and new hard tissue formation inside the root canal [82]. In addition, preparation of PRP involves additional steps including drawing a blood sample from the patient and centrifuging the blood with an anticoagulant. Behavior management of young children who constitute the majority of patients for regenerative endodontic procedures, can therefore be an obstacle for clinical utilization of PRP [73]. 

In 1976, collagen-calcium phosphate gel was investigated to close the apex of pulpless open apex teeth as an alternative to calcium hydroxide apexification [83]. The result showed that teeth filled with collagen-calcium phosphate gel appeared to have connective tissue in growth and various forms of hard tissue lining the root canal walls. The interesting point of this study was that they did not add any stem/progenitor cells (neither transplantation nor cell homing approach) or growth factors to their scaffold, which are components of the tissue engineering triad. Collagen has been used as a scaffold in several *in vivo* experiments based on the tissue engineering triad [84, 85]. The triad of DPSCs, collagen scaffold, and Dentin Matrix Protein (DMP)-1 were placed in simulated perforation sites in dentin slices [84]. After 6 weeks of subcutaneous implantation on the dorsal surfaces of immuno-deficient mice, organized matrix formation similar to that of pulpal tissue was seen which might lead to hard tissue formation. In the group using only collagen scaffold, degrading collagen was observed without the presence of any calcified tissues [84]. Collagen gel solution was successfully used in a cell-homing approach to regeneration along with multiple combined growth factors such as *beta-Fibroblast Growth Factor* (bFGF), *Vascular Endothelial Growth Factor* (VEGF), or *Platelet-Derived Growth Factor* (PDGF) with a basal set of *Nerve Growth Factor* (NGF) and *Bone Morphogenetic Protein-7* (BMP7) [85]. 

## Potential role of growth factors

The proliferation, differentiation, survival and motility of DPSCs are regulated by a network composed of various signaling molecules, receptors and transcription control systems in the pulp tissue. Multiple growth factors, including BMPs, VEGF and *Transforming Growth Factor beta* (TGFβs), have been implicated in mediating these biological processes and they can play potentially important roles in pulp regeneration. It is necessary to integrate different signaling pathways into a regulatory network to precisely orchestrate gene expression. Therefore, an understanding of each growth factor and its relationship with dental pulp stem cells is an indispensable step in developing a predictable pulp regeneration protocol.

***Bone morphogenetic proteins (BMPs)***

BMP was discovered by Urist in 1965 who showed that demineralized lyophilized segments of rabbit bone induce new bone in intramuscular sites [86]. Subsequently, BMPs were isolated from adult bone matrix in mammals by extracting demineralized bone matrix.

The human genome encodes 20 BMPs and this BMP family can be divided into four distinct subfamilies: *first*, BMP2 and 4; *second*, BMP3 and BMP3B; *third*, BMPs 5, 6, 7 and 8; and *fourth*, Growth Differentiation Factors (GDFs) 5, 6 and 7, also known as cartilage-derived morphogenetic proteins* 1, 2* and *3* [87]*.*

BMPs are related to the superfamily of TGFβ group and their ability to induce formation of bone and cartilage is well known [88]. In response to treatment with recombinant human BMP2, pulp-derived mesenchymal cells differentiate into dentin-forming pre-odontoblasts [89]. When combined with total EDTA-soluble fraction of dentin, recombinant human BMP2 along with TGFβ1 also stimulates odontoblast differentiation in organ cultures of dental papilla cells [90]. 

When used as a pulp capping material over amputated canine pulp in an *in vivo* model, human recombinant BMP2 or BMP4 induces dentin formation [91]. In this study, human recombinant BMP2 or BMP4 were added to dentin matrix power, chondroitin 6-sulfate sodium salt, or type 1 rat tail collagen, as carriers. After two months, the amputated pulp was filled with tubular dentin in the lower part. However, when BMP was delivered using reconstituted type I collagen matrix, no distinct tubular dentine was formed, unlike an earlier experiment in which BMP2 or BMP4 was implanted with enriched, inactivated dentine matrix [92]. These findings suggest that both BMP2 and BMP4 induce osteodentine formation if combined with collagen matrix. However, some other matrix components sequestered in inactivated dentine matrix might be essential for further differentiation into odontoblasts. Autologous transplantation of BMP11-transfected cells cultured as a pellet stimulated reparative dentin formation on amputated pulp in a dog model [93].

***Vascular endothelial growth factor (VEGF)***

The tissue engineering triad of scaffold, stem cells and morphogens might be essential for successful pulp regeneration. However, without proper angiogenesis/vasculogenesis to supply enough nutrients and oxygen to the transplanted or migrated stem cells into the root canal, pulp regeneration would not be successful. VEGF is an excellent regulator of angiogenesis and is known to increase vascular permeability.

VEGF is a potent endothelial cell mitogen and angiogenic factor that has been shown to play a central role in vascular responses that accompany a number of physiological and pathological processes [94]. VEGF is produced by a variety of cell types, including keratinocytes, macrophages, mast cells, smooth muscle cells and several types of tumors [94].

VEGF induces chemotaxis, proliferation and differentiation of human dental pulp cells [95, 96]. In addition, VEGF is also involved in the proliferation of endothelial cells, enhances their survival in the toxic oxygen-deficient environment, and stimulates neovascularization in the area of injury [97]. In an *in vivo* study using the immunodeficient mice model, tooth slices treated with 0 or 50 ng/mL rhVEGF165 for one hour, demonstrated increased pulp microvessel density, which is potentially beneficial for pulp regeneration [98].

Dentin contains many growth factors including VEGF and cytokines that are embedded in the matrix during dentinogenesis through their interactions with non-collagenous proteins and other extracellular matrix components [99]. Among these growth factors, angiogenic growth factors (VEGF) released from the matrix as a result of matrix breakdown during tissue injury or during pulp regeneration procedures by EDTA, could make an important contribution to the overall reparative/regenerative response of the dentine-pulp complex [100]. According to Zhang’s study, dentin matrix components showed a dose-dependent response in the expression of the proangiogenic growth factor VEGF and its receptor VEGFR2 [101]. These proangiogenic effects of relatively low concentrations of dentin matrix components could be associated with revascularization during pulp repair and regeneration.

***Transforming growth factor beta (TGFβ)***

Presence of TGFβ has been identified in developing teeth and human dentin. With respect to its role in pulp repair, TGFβ1 was shown to stimulate type I collagen synthesis, alkaline phosphatase activity, and in culture proliferation of mammalian pulp cells [102, 103]. Results of a study by Melin *et al. *also suggested that TGFβ1 could be directly involved in the regulation of cell proliferation, migration, and extracellular matrix production in human dental pulp [104]. Together with FGF2, TGFβ1 stimulated the odontoblastic differentiation of STRO-1^+^ DPSCs *in vitro *with expression of dentin sialoprotein (DSP) and dentin matrix protein (DMP)-1 mRNA [105].

TGFβ1 has been shown to be chemotactic for dental papilla-derived cells as well as for other cell types [106]. At the stage of cell recruitment, this chemotactic property of TGFβ1 could attract the stem/progenitor cells to the site of tissue injury in the tooth.

The aforementioned growth factors could be incorporated in tissue engineering scaffolds and delivered into the root canal to promote pulp regeneration. However, it is also been demonstrated that dentine matrix itself contains a broad spectrum of growth factors including TGFβ, VEGF, BMPs, FGF2, and Epidermal Growth Factor (EGF) which stimulate and modulate many of the cellular events taking place during pulp regeneration [100]. During the pulp revascularization procedure with EDTA application and induction of blot clot, growth factors would be released from the dentin matrix and platelets. However, different concentrations of the same molecules can show a spectrum of effects ranging from inflammation or stimulation of regeneration to cell apoptosis [107].

In a recent interesting study, a cell-homing approach using growth factors was proposed to regenerate the pulp-dentin complex rather than transplantation of stem/progenitor cells in entire human teeth [85]. This study showed cellularized and vascularized tissues and new dentin formation over the surface of dentinal wall in endodontically treated human teeth implanted in mouse dorsum. They produced dental pulp-like tissue in the entire root canal by using combined delivery of bFGF, VEGF, or PDGF, NGF and BMP7. The cell homing strategy avoids the *in vitro* culture of autologous cells and therefore it could be a simple, practical and economical approach for pulp regeneration [108].

## Conclusion

The current protocol for pulp regeneration shows promising results in terms of healing of periapical lesions and survival rate of treated teeth compared to traditional methods. However, there are several problems with the protocol and the outcomes are unpredictable. Therefore, every step in the treatment protocol should be revised to attain a more biocompatible strategy. More importantly, the effect of adding tissue-engineering triad components (stem cells, bioactive scaffolds and growth factors) to the current protocol needs to be studied in more relevant in situ animal models with immature teeth and concurrent pulp necrosis.

## References

[1] Cvek M (1992). prognosis of luxated non-vital maxillary incisors treated with calcium hydroxide and filled with gutta percha Endod. Dent Traumatol.

[2] Rafter M (2005). Apexification : A review. Dent Traumatol.

[3] Sheehy E, Roberts G (1997). Use of calcium hydroxide for apical barrier formation and healing in non-vital immature permanent teeth: a review. Br Dent J.

[4] Andreasen J, Farik B, Munksgaard E (2002). Long-term calcium hydroxide as a root canal dressing may increase risk of root fracture. Dent Traumatol.

[5] Torabinejad M, Chivian N (1999). Clinical applications of mineral trioxide aggregate. J Endod.

[6] Bogen G, Kuttler S (2009). Mineral trioxide aggregate obturation: a review and case series. J Endod.

[7] Nosrat A, Asgary S, Eghbal MJ, Ghoddusi J, Bayat-Movahed S (2011). Calcium-enriched mixture cement as artificial apical barrier: A case series. J Conserv Dent.

[8] Witherspoon D, Small J, Regan J, Nunn M (2008). Retrospective Analysis of Open Apex Teeth Obturated with Mineral Trioxide Aggregate. J Endod.

[9] Mente J, Hage N, Pfefferle T, Koch M, Dreyhaupt J, Staehle H, Friedman S (2009). Mineral Trioxide Aggregate Apical Plugs in Teeth with Open Apical Foramina: A Retrospective Analysis of Treatment Outcome. J Endod.

[10] Holden D, Schwartz S, Kirkpatrick T, Schindler W (2008). Clinical Outcomes of Artificial Root-end Barriers with Mineral Trioxide Aggregate in Teeth with Immature Apices. J Endod.

[11] Hargreaves K, Geisler T, Henry M, Wang Y (2008). Regeneration potential of the young permanent tooth: What does the future hold. J Endod.

[12] Hargreaves KM, Diogenes A, Teixeira FB (2013). Treatment options: biological basis of regenerative endodontic procedures. J Endod.

[13] Huang G, Sonoyama W, Liu Y (2008). The hidden treasure in apical papilla: the potential role in pulp/dentin regeneration and bioroot engineering. J Endod.

[14] (2013). Considerations for a Regenerative Endodontics Procedure.

[15] Reynolds K, Johnson J, Cohenca N (2009). Pulp revascularization of necrotic bilateral bicuspids using a modified novel technique to eliminate potential coronal discolouration: a case report. Int Endod J.

[16] Shin S, Albert J, Mortman R (2009). One step pulp revascularization treatment of an immature permanent tooth with chronic apical abscess: a case report. Int Endod J.

[17] Banchs F, Trope M (2004). Revascularization of immature permanent teeth with apical periodontitis: new treatment protocol?. J Endod.

[18] Petrino J, Boda K, Shambarger S, Bowles W, McClanahan S (2010). Challenges in regenerative endodontics: a case series. J Endod.

[19] Ding R, Cheung G, Chen J, Yin X, Wang Q, Zhang C (2009). Pulp Revascularization of Immature Teeth With Apical Periodontitis: A Clinical Study. J Endod.

[20] Chueh L, Huang G (2006). Immature Teeth With Periradicular Periodontitis or Abscess Undergoing Apexogenesis: A Paradigm Shift. J Endod.

[21] Shah N, Logani A, Bhaskar U, Aggarwal V (2008). Efficacy of Revascularization to Induce Apexification/Apexogensis in Infected, Nonvital, Immature Teeth: A Pilot Clinical Study. J Endod.

[22] Chueh L, Ho Y, Kuo T, Lai W, Chen Y, Chiang C (2009). Regenerative endodontic treatment for necrotic immature permanent teeth. J Endod.

[23] Nosrat A, Seifi A, Asgary S (2011). Regenerative endodontic treatment (revascularization) for necrotic immature permanent molars: a review and report of two cases with a new biomaterial. J Endod.

[24] Cehreli ZC, Isbitiren B, Sara S, Erbas G (2011). Regenerative endodontic treatment (revascularization) of immature necrotic molars medicated with calcium hydroxide: a case series. J Endod.

[25] Windley W, Teixeira F, Levin L, Sigurdsson A, Trope M (2005). Disinfection of immature teeth with a triple antibiotic paste. J Endod.

[26] Hoshino E, Kurihara-Ando N, Sato I, Uematsu H, Sato M, Kota K, Iwaku M (1996). In-vitro antibacterial susceptibility of bacteria taken from infected root dentine to a mixture of ciprofloxacin, metronidazole and minocycline. Int Endod J.

[27] Adl A, Shojaee NS, Motamedifar M (2012). A Comparison between the antimicrobial effects of triple antibiotic paste and calcium hydroxide against Entrococcus Faecalis. Iran Endod J.

[28] Iwaya S, Ikawa M, Kubota M (2001). Revascularization of an immature permanent tooth with apical periodontitis and sinus tract. Dent Traumatol.

[29] Thibodeau B, Trope M (2007). Pulp revascularization of a necrotic infected immature permanent tooth: case report and review of the literature. Pediatr Dent.

[30] Nosrat A, Homayounfar N, Oloomi K (2012). Drawbacks and unfavorable outcomes of regenerative endodontic treatments of necrotic immature teeth: a literature review and report of a case. J Endod.

[31] Graham L, Cooper PR, Cassidy N, Nor JE, Sloan AJ, Smith AJ (2006). The effect of calcium hydroxide on solubilisation of bio-active dentine matrix components. Biomaterials.

[32] Lovelace TW, Henry MA, Hargreaves KM, Diogenes A (2011). Evaluation of the delivery of mesenchymal stem cells into the root canal space of necrotic immature teeth after clinical regenerative endodontic procedure. J Endod.

[33] Torabinejad M, Turman M (2011). Revitalization of tooth with necrotic pulp and open apex by using platelet-rich plasma: a case report. J Endod.

[34] Jadhav G, Shah N, Logani A (2012). Revascularization with and without platelet-rich plasma in nonvital, immature, anterior teeth: a pilot clinical study. J Endod.

[35] Wigler R, Kaufman AY, Lin S, Steinbock N, Hazan-Molina H, Torneck CD (2013). Revascularization: a treatment for permanent teeth with necrotic pulp and incomplete root development. J Endod.

[36] Bose R, Nummikoski P, Hargreaves K (2009). A retrospective evaluation of radiographic outcomes in immature teeth with necrotic root canal systems treated with regenerative endodontic procedures. J Endod.

[37] Jeeruphan T, Jantarat J, Yanpiset K, Suwannapan L, Khewsawai P, Hargreaves KM (2012). Mahidol study 1: comparison of radiographic and survival outcomes of immature teeth treated with either regenerative endodontic or apexification methods: a retrospective study. J Endod.

[38] Kim J, Kim Y, Shin S, Park J, Jung I (2010). Tooth discoloration of immature permanent incisor associated with triple antibiotic therapy: a case report. J Endod.

[39] Lenherr P, Allgayer N, Weiger R, Filippi A, Attin T, Krastl G (2012). Tooth discoloration induced by endodontic materials: a laboratory study. Int Endod J.

[40] Nosrat A, Asgary S (2010). Apexogenesis treatment with a new endodontic cement: a case report. J Endod.

[41] Asgary S, Ehsani S (2013). Periradicular surgery of human permanent teeth with calcium-enriched mixture cement. Iran Endod J.

[42] Moradi S, Disfani R, Ghazvini K, Lomee M (2013). Sealing Ability of Orthograde MTA and CEM Cement in Apically Resected Roots Using Bacterial Leakage Method. Iran Endod J.

[43] Yavari HR, Samiei M, Shahi S, Aghazadeh M, Jafari F, Abdolrahimi M, Asgary S (2012). Microleakage comparison of four dental materials as intra-orifice barriers in endodontically treated teeth. Iran Endod J.

[44] Chen MY, Chen KL, Chen CA, Tayebaty F, Rosenberg PA, Lin LM (2012). Responses of immature permanent teeth with infected necrotic pulp tissue and apical periodontitis/abscess to revascularization procedures. Int Endod J.

[45] Fouad AF (2011). The microbial challenge to pulp regeneration. Adv Dent Res.

[46] Kakoli P, Nandakumar R, Romberg E, Arola D, Fouad AF (2009). The effect of age on bacterial penetration of radicular dentin. J Endod.

[47] Lenzi R, Trope M (2012). Revitalization procedures in two traumatized incisors with different biological outcomes. J Endod.

[48] Thibodeau B, Teixeira F, Yamauchi M, Caplan D, Trope M (2007). Pulp Revascularization of Immature Dog Teeth With Apical Periodontitis. J Endod.

[49] Wang X, Thibodeau B, Trope M, Lin L, Huang G (2010). Histologic characterization of regenerated tissues in canal space after the revitalization/revascularization procedure of immature dog teeth with apical periodontitis. J Endod.

[50] da Silva L, Nelson-Filho P, da Silva R, Flores D, Heilborn C, Johnson J, Cohenca N (2010). Revascularization and periapical repair after endodontic treatment using apical negative pressure irrigation versus conventional irrigation plus triantibiotic intracanal dressing in dogs’ teeth with apical periodontitis. Oral Surg Oral Med Oral Pathol Oral Radiol Endod.

[51] Yamauchi N, Yamauchi S, Nagaoka H, Duggan D, Zhong S, Lee SM, Teixeira FB, Yamauchi M (2011). Tissue engineering strategies for immature teeth with apical periodontitis. J Endod.

[52] Torabinejad M, Faras H (2012). A clinical and histological report of a tooth with an open apex treated with regenerative endodontics using platelet-rich plasma. J Endod.

[53] Shimizu E, Jong G, Partridge N, Rosenberg PA, Lin LM (2012). Histologic observation of a human immature permanent tooth with irreversible pulpitis after revascularization/regeneration procedure. J Endod.

[54] Martin G, Ricucci D, Gibbs JL, Lin LM (2013). Histological Findings of Revascularized/Revitalized Immature Permanent Molar with Apical Periodontitis Using Platelet-rich Plasma. J Endod.

[55] Trevino EG, Patwardhan AN, Henry MA, Perry G, Dybdal-Hargreaves N, Hargreaves KM, Diogenes A (2011). Effect of irrigants on the survival of human stem cells of the apical papilla in a platelet-rich plasma scaffold in human root tips. J Endod.

[56] Ring K, Murray P, Namerow K, Kuttler S, Garcia-Godoy F (2008). The comparison of the effect of endodontic irrigation on cell adherence to root canal dentin. J Endod.

[57] Casagrande L, Demarco FF, Zhang Z, Araujo FB, Shi S, Nor JE (2010). Dentin-derived BMP-2 and odontoblast differentiation. J Dent Res.

[58] Ruparel NB, Teixeira FB, Ferraz CC, Diogenes A (2012). Direct effect of intracanal medicaments on survival of stem cells of the apical papilla. J Endod.

[59] Yamamura T (1985). Differentiation of pulpal cells and inductive influences of various matrices with reference to pulpal wound healing. J Dent Res.

[60] Barry FP (2003). Biology and clinical applications of mesenchymal stem cells. Birth defects research. Part C, Embryo today: reviews.

[61] Sonoyama W, Liu Y, Fang D, Yamaza T, Seo B, Zhang C, Liu H, Gronthos S, Wang C, Shi S (2006). Mesenchymal stem cell-mediated functional tooth regeneration in Swine. PLoS One.

[62] Gronthos S, Brahim J, Li W, Fisher LW, Cherman N, Boyde A, DenBesten P, Robey PG, Shi S (2002). Stem cell properties of human dental pulp stem cells. J Dent Res.

[63] Gronthos S, Mankani M, Brahim J, Robey PG, Shi S (2000). Postnatal human dental pulp stem cells (DPSCs) in vitro and in vivo. Proc Natl Acad Sci USA.

[64] Huang GT, Shagramanova K, Chan SW (2006). Formation of odontoblast-like cells from cultured human dental pulp cells on dentin in vitro. J Endod.

[65] Huang GT, Sonoyama W, Chen J, Park SH (2006). In vitro characterization of human dental pulp cells: various isolation methods and culturing environments. Cell Tissue Res.

[66] Alongi DJ, Yamaza T, Song Y, Fouad AF, Romberg EE, Shi S, Tuan RS, Huang GT (2010). Stem/progenitor cells from inflamed human dental pulp retain tissue regeneration potential. Regen Med.

[67] Isaka J, Ohazama A, Kobayashi M, Nagashima C, Takiguchi T, Kawasaki H, Tachikawa T, Hasegawa K (2001). Participation of periodontal ligament cells with regeneration of alveolar bone. J Periodontol.

[68] McCulloch CA, Bordin S (1991). Role of fibroblast subpopulations in periodontal physiology and pathology. J Periodontal Res.

[69] Gay IC, Chen S, MacDougall M (2007). Isolation and characterization of multipotent human periodontal ligament stem cells. Orthod Craniofac Res.

[70] Xu J, Wang W, Kapila Y, Lotz J, Kapila S (2009). Multiple differentiation capacity of STRO-1+/CD146+ PDL mesenchymal progenitor cells. Stem Cells Dev.

[71] Lindroos B, Maenpaa K, Ylikomi T, Oja H, Suuronen R, Miettinen S (2008). Characterisation of human dental stem cells and buccal mucosa fibroblasts. Biochem Biophys Res Commun.

[72] Yamauchi N, Nagaoka H, Yamauchi S, Teixeira FB, Miguez P, Yamauchi M (2011). Immunohistological characterization of newly formed tissues after regenerative procedure in immature dog teeth. J Endod.

[73] Verma P, Fouad A (2012). What is the Public Health Need for Regenerative Endodontics. J Endod.

[74] Nakashima M, Iohara K (2011). Regeneration of dental pulp by stem cells. Adv Dent Res.

[75] Zheng Y, Wang XY, Wang YM, Liu XY, Zhang CM, Hou BX, Wang SL (2012). Dentin regeneration using deciduous pulp stem/progenitor cells. J Dent Res.

[76] Zhu W, Zhu X, Huang GT, Cheung GS, Dissanayaka WL, Zhang C (2013). Regeneration of dental pulp tissue in immature teeth with apical periodontitis using platelet-rich plasma and dental pulp cells. Int Endod J.

[77] Aggarwal S, Pittenger MF (2005). Human mesenchymal stem cells modulate allogeneic immune cell responses. Blood.

[78] Nasef A, Ashammakhi N, Fouillard L (2008). Immunomodulatory effect of mesenchymal stromal cells: possible mechanisms. Regen Med.

[79] Iohara K, Imabayashi K, Ishizaka R, Watanabe A, Nabekura J, Ito M, Matsushita K, Nakamura H, Nakashima M (2011). Complete pulp regeneration after pulpectomy by transplantation of CD105+ stem cells with stromal cell-derived factor-1. Tissue Eng Part A.

[80] Ostby BN (1961). The role of the blood clot in endodontic therapy An experimental histologic study. Acta Odontol Scand.

[81] Sonoyama W, Liu Y, Yamaza T, Tuan R, Wang S, Shi S, Huang G (2008). Characterization of the apical papilla and its residing stem cells from human immature permanent teeth: a pilot study. J Endod.

[82] Zhu X, Zhang C, Huang GT, Cheung GS, Dissanayaka WL, Zhu W (2012). Transplantation of dental pulp stem cells and platelet-rich plasma for pulp regeneration. J Endod.

[83] Nevins AJ, Finkelstein F, Borden BG, Laporta R (1976). Revitalization of pulpless open apex teeth in rhesus monkeys, using collagen-calcium phosphate gel. J Endod.

[84] Prescott RS, Alsanea R, Fayad MI, Johnson BR, Wenckus CS, Hao J, John AS, George A (2008). In vivo generation of dental pulp-like tissue by using dental pulp stem cells, a collagen scaffold, and dentin matrix protein 1 after subcutaneous transplantation in mice. J Endod.

[85] Kim JY, Xin X, Moioli EK, Chung J, Lee CH, Chen M, Fu SY, Koch PD, Mao JJ (2010). Regeneration of dental-pulp-like tissue by chemotaxis-induced cell homing. Tissue Eng Part A.

[86] Urist MR (1965). Bone: formation by autoinduction. Science.

[87] Reddi AH (1998). Role of morphogenetic proteins in skeletal tissue engineering and regeneration. Nat Biotechnol.

[88] Wang EA, Rosen V, D'Alessandro JS, Bauduy M, Cordes P, Harada T, Israel DI, Hewick RM, Kerns KM, LaPan P (1990). Recombinant human bone morphogenetic protein induces bone formation. Proc Natl Acad Sci U S A.

[89] Nakashima M, Nagasawa H, Yamada Y, Reddi AH (1994). Regulatory role of transforming growth factor-beta, bone morphogenetic protein-2, and protein-4 on gene expression of extracellular matrix proteins and differentiation of dental pulp cells. Dev Biol.

[90] Begue-Kirn C, Smith AJ, Ruch JV, Wozney JM, Purchio A, Hartmann D, Lesot H (1992). Effects of dentin proteins, transforming growth factor beta 1 (TGF beta 1) and bone morphogenetic protein 2 (BMP2) on the differentiation of odontoblast in vitro. Int J Dev Biol.

[91] Nakashima M (1994). Induction of dentin formation on canine amputated pulp by recombinant human bone morphogenetic proteins (BMP)-2 and -4. J Dent Res.

[92] Nakashima M (1994). Induction of dentine in amputated pulp of dogs by recombinant human bone morphogenetic proteins-2 and -4 with collagen matrix. Arch Oral Biol.

[93] Nakashima M, Iohara K, Ishikawa M, Ito M, Tomokiyo A, Tanaka T, Akamine A (2004). Stimulation of reparative dentin formation by ex vivo gene therapy using dental pulp stem cells electrotransfected with growth/differentiation factor 11 (Gdf11). Hum Gene Ther.

[94] Ferrara N, Bunting S (1996). Vascular endothelial growth factor, a specific regulator of angiogenesis. Curr Opin Nephrol Hypertens.

[95] Artese L, Rubini C, Ferrero G, Fioroni M, Santinelli A, Piattelli A (2002). Vascular endothelial growth factor (VEGF) expression in healthy and inflamed human dental pulps. J Endod.

[96] Matsushita K, Motani R, Sakuta T, Yamaguchi N, Koga T, Matsuo K, Nagaoka S, Abeyama K, Maruyama I, Torii M (2000). The role of vascular endothelial growth factor in human dental pulp cells: induction of chemotaxis, proliferation, and differentiation and activation of the AP-1-dependent signaling pathway. J Dent Res.

[97] Nor JE, Christensen J, Mooney DJ, Polverini PJ (1999). Vascular endothelial growth factor (VEGF)-mediated angiogenesis is associated with enhanced endothelial cell survival and induction of Bcl-2 expression. Am J Pathol.

[98] Mullane EM, Dong Z, Sedgley CM, Hu JC, Botero TM, Holland GR, Nor JE (2008). Effects of VEGF and FGF2 on the revascularization of severed human dental pulps. J Dent Res.

[99] Smith AJ (2003). Vitality of the dentin-pulp complex in health and disease: growth factors as key mediators. J Dent Educ.

[100] Roberts-Clark DJ, Smith AJ (2000). Angiogenic growth factors in human dentine matrix. Arch Oral Biol.

[101] Zhang R, Cooper PR, Smith G, Nor JE, Smith AJ (2011). Angiogenic activity of dentin matrix components. J Endod.

[102] Liang RF, Nishimura S, Sato S (1992). Effects of epidermal growth factor and transforming growth factor-beta on insulin-induced differentiation in rat dental pulp cells. Arch Oral Biol.

[103] Nakashima M (1992). The effects of growth factors on DNA synthesis, proteoglycan synthesis and alkaline phosphatase activity in bovine dental pulp cells. Arch Oral Biol.

[104] Melin M, Joffre-Romeas A, Farges JC, Couble ML, Magloire H, Bleicher F (2000). Effects of TGFbeta1 on dental pulp cells in cultured human tooth slices. J Dent Res.

[105] He H, Yu J, Liu Y, Lu S, Liu H, Shi J, Jin Y (2008). Effects of FGF2 and TGFbeta1 on the differentiation of human dental pulp stem cells in vitro. Cell Biol Int.

[106] Kwon SM, Kim SA, Yoon JH, Ahn SG (2010). Transforming growth factor beta1-induced heat shock protein 27 activation promotes migration of mouse dental papilla-derived MDPC-23 cells. J Endod.

[107] Cooper PR, Takahashi Y, Graham LW, Simon S, Imazato S, Smith AJ (2010). Inflammation-regeneration interplay in the dentine-pulp complex. J Dent.

[108] Mao JJ, Stosich MS, Moioli EK, Lee CH, Fu SY, Bastian B, Eisig SB, Zemnick C, Ascherman J, Wu J, Rohde C, Ahn J (2010). Facial reconstruction by biosurgery: cell transplantation versus cell homing. Tissue Eng Part B Rev.

